# Inborn errors of immunity (primary immunodeficiencies)

**DOI:** 10.1186/s13223-024-00938-z

**Published:** 2025-01-08

**Authors:** Vy H. D. Kim, Julia E. M. Upton, Beata Derfalvi, Kyla J. Hildebrand, Christine McCusker

**Affiliations:** 1 Division of Immunology and Allergy, Department of Pediatrics, The Hospital for Sick Children, Temerty School of Medicine, University of Toronto, Toronto, ON Canada; 2 Division of Clinical Immunology and Allergy, Department of Medicine, Temerty School of Medicine, University of Toronto, Toronto, ON Canada; 3 Division of Immunology, IWK Health Centre, Department of Pediatrics, Dalhousie University, Halifax, NS Canada; 4 Division of Immunology, Department of Pediatrics, BC Children’s Hospital, Vancouver, BC Canada; 5 BC Children’s Hospital Research Institute, Vancouver, BC Canada; 6 Division of Allergy and Clinical Immunology, Department of Pediatrics, Montreal Children’s Hospital, McGill University Health Centre, Montreal, QC Canada

## Abstract

Primary immunodeficiencies (PID), now often referred to as inborn errors of immunity (IEI), are a large heterogeneous group of disorders that result from deficiencies in immune system development and/or function. IEIs can be broadly classified as disorders of adaptive immunity (e.g., combined or humoral immunodeficiencies) or of innate immunity (e.g., phagocyte and complement disorders). Although the clinical manifestations of IEIs are highly variable, traditionally many disorders involve an increased susceptibility to infection. Research in recent years has underscored how IEI can present with features other than infection such as: severe atopy, autoimmunity, autoinflammation, lymphoproliferation, and/or malignancy resulting from immune dysregulation. Early consultation with a clinical immunologist is essential, as timely diagnosis and treatment are imperative for preventing significant disease-associated morbidity and mortality. The treatment of IEIs is complex and generally requires both supportive and definitive strategies, including but not limited to, immunoglobulin replacement therapy, antibiotic prophylaxis, immune response modifiers, and hematopoietic stem cell transplantation. This article provides an overview of the major categories of IEIs and strategies for the appropriate diagnosis and management of these disorders.

**Supplementary Information:**

The online version contains supplementary material available at 10.1186/s13223-024-00938-z.

## Key take-home messages

- IEIs are more frequent than previously believed, with an estimated prevalence of 1 in 1200 live births.
- Clinical presentation is highly variable, but most disorders involve an increased susceptibility to infection. Excessive inflammatory responses and autoimmunity are also common manifestations of immune deficiency.
- IEIs should be suspected at any age in patients with: recurrent, severe and/or unusual infection(s); poor response to use of appropriate antibiotics; failure to thrive; persistent thrush or skin abscesses; or a family history of IEI. Multiple hypersensitivities and atopy may also signal an IEI.
- Consultation with a clinical immunologist is required to confirm the diagnosis of IEI and to establish an appropriate treatment plan. Genetic testing is routinely utilized as a genetic diagnosis and knowledge of the specific immune defect can guide targeted treatment and personalized care.
- SCID is fatal unless the underlying defect is corrected and, therefore, definitive therapy with HSCT should be initiated as quickly as possible. The importance of prompt diagnosis has led to newborn screening for SCID using the TREC assay, an effective screen for some types of SCID but not other IEIs.
- Ig replacement therapy is the mainstay of therapy for antibody-deficiency disorders. It is also an important supportive treatment for many patients with other forms of IEI including SCID/CID. Several Ig replacement products are available in Canada.
- Antibiotic and antifungal prophylaxis, and optimized immunization schedules are also recommended for many IEIs to prevent the frequency and severity of infections.

**Supplementary Information:**

The online version contains supplementary material available at 10.1186/s13223-024-00938-z.

## Introduction

Primary immunodeficiencies (PID), now often referred to as inborn errors of immunity (IEI), are a heterogeneous group of disorders characterized by poor or absent function in one or more components of the immune system. Affected individuals are predisposed to increased frequency and severity of infection, autoimmunity, aberrant inflammation including atopy, and/or malignancy. Close to 700 different disorders have been genetically identified to date, with new disorders continually being recognized [1]. Most IEIs result from germline gene variants that affect immune system development and/or function; however, acquired forms can also occur called “phenocopies of IEI” [2, 3], such as neutralizing anti-interferon-γ autoantibody-associated immunodeficiency (which has been noted in over 95% of patients with disseminated infections by nontuberculous mycobacteria) [3] and autoantibodies against type 1 interferons, which have been associated with severe life-threatening infections from severe acute respiratory syndrome coronavirus 2 (SARS-CoV-2) [4]. When assessing for IEI, it is important to consider secondary causes of immunodeficiency, such as viral or bacterial infections, malnutrition, immunoglobulin (Ig) loss, malignancy or treatment with drugs that induce immunosuppression (see *Secondary Immunodeficiency* article in this supplement) [5, 6].

With the exception of selective immunoglobulin A (IgA) deficiency and mannose binding lectin (MBL) deficiency, the estimated overall prevalence of IEI in the United States is approximately 1 in 1200 live births [7]. Selective IgA deficiency occurs in approximately 1 in 300 to 1 in 500 persons [7]. While MBL deficiency has been identified in 5–7% of Caucasian populations, its clinical impact is debated [8].

The clinical presentation of IEIs is highly variable. Many disorders involve increased susceptibility to infection. In fact, many IEIs present as “routine” infections (often of the sinuses, ears and lungs) and, therefore, may initially go undetected in the primary-care setting. Serious infections can result in hospital admissions for investigations and management. IEIs may present at any age, and the accurate and timely diagnosis of these disorders requires a high index of suspicion and specialized testing. Therefore, consultation with a clinical immunologist who is experienced in the evaluation and management of IEI is essential, as early diagnosis, treatment and targeted immunizations to prevent infections are critical for preventing significant disease-associated morbidity and mortality and improving patient outcomes [9, 10]. This article provides an overview of the major categories of IEIs as well as strategies for the timely identification, diagnosis, and management of these disorders.

## Classification

IEIs are broadly classified according to the component of the immune system that is primarily disrupted: adaptive or innate immunity (see *Introduction to Immunology and Immune Disorders* in this supplement for more information on adaptive and innate immunity). Disorders of immune dysregulation include syndromes that are associated with autoimmunity, hyperinflammation, and immune dysregulation as predominant features, rather than an overt pathological risk of infections [1, 11]. Primary atopic disorders are a group of monogenic diseases where the predominant features are pathogenic allergic- or atopic effector-related symptoms [12]. Table 1 presents a simplified classification highlighting the major categories of IEIs [1, 2, 9, 13, 14].

**Table 1:**
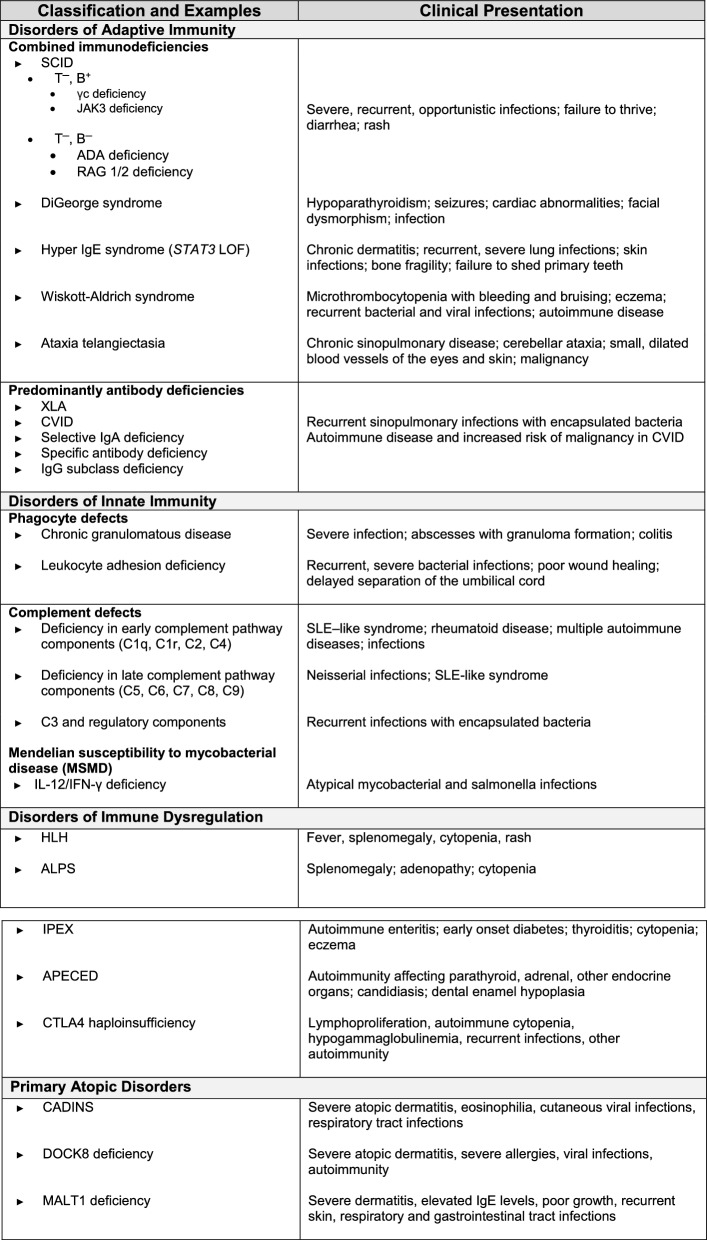
Simplified classification of IEIs: examples and typical clinical presentations

ADA, adenosine deaminase; AIRE: autoimmune regulator; ALPS, autoimmune lymphoproliferative syndrome; APECED, autoimmune polyendocrinopathy candidiasis and ectodermal dystrophy; CADINS, CARD11-associated atopy with dominant interference of NF-κB signaling; CVID, common variable immunodeficiency; HLH, hemophagocytic lymphohistiocytosis; IgA, immunoglobulin A; IgE, immunoglobulin E; IgG, immunoglobulin G; IFNγ, interferon-gamma; IL, interleukin; IPEX, immune dysregulation polyendocrinopathy enteropathy X-linked; JAK3, Janus kinase 3; LOF, loss of function; RAG, recombination activating gene; SCID, severe combined immunodeficiency; SLE, systemic lupus erythematosus; XLA, X-linked agammaglobulinemia

### Disorders of adaptive immunity

T-cells and B-cells are the primary cells of the adaptive immune system. B-cells mediate antibody production and, therefore, play a major role in antibody-mediated (humoral) immunity. Defects relating to B-cell development and/or maturation result in humoral (antibody-deficiency) disorders. T-cells, on the other hand, govern cell-mediated immune responses. Defects occurring at any stage of T-cell development, differentiation, and maturation lead to cellular defects. Since B-cell-mediated antibody production requires intact T-cell function, most T-cell defects can also involve antibody deficiency. Hence, disorders involving T-cells are termed combined immunodeficiencies (CID) [2, 9].

### Disorders of innate immunity

Innate immune responses represent the first line of defense against potential pathogens. Appropriate recognition of threats and induction of the inflammatory cascade are essential steps in the removal of these organisms from the system. Failure of the innate system to identify pathogens delays the induction of the immune response and may worsen outcomes of infection.

Numerous cells and proteins are involved in the innate immune response including toll-like receptors (TLR), phagocytes (neutrophils and macrophages), dendritic cells, and complement proteins. TLR recognize foreign proteins such as those unique to infectious organisms. Phagocytes are primarily responsible for phagocytosis, the process by which cells engulf and eliminate invading pathogens. Complement proteins function to identify and opsonize (coat) foreign antigens, rendering them susceptible to phagocytosis. Defects in the development and function of any of these elements of innate immunity may lead to IEIs.

### Disorders of immune dysregulation

The immune system has several checkpoints in place to prevent autoimmunity and to control the immune response. Control mechanisms that limit autoreactive T-cells occur through central and peripheral tolerance [15–17]. Central T-cell tolerance takes place in the thymus through processes whereby newly formed T-cells that are strongly self-reactive are eliminated (negative selection) or undergo differentiation into regulatory T-cells (Treg). Treg cells play key roles in mediating peripheral tolerance, which takes place in the immune periphery (i.e., outside the thymus) [18]. Treg cells express the transcription factor FoxP3 and can suppress T-cell responses to limit the immune response and prevent autoimmunity. Defects affecting the checkpoints or tolerance mechanisms lead to immune dysregulation, which can manifest as autoimmunity, allergy, inflammation, lymphoproliferation and/or malignancy [19–21].

### Primary atopic disorders

Primary atopic disorders (PAD) are a group of monogenic diseases characterized by predominant allergy or atopy-related symptoms. Patients can have severe, difficult to control, and multiple allergic disease, with or without associated immunodeficiency. Several altered processes have been implicated in PAD, including altered cytokine signaling, skin barrier disruption, mast cell dysfunction, impaired T-cell and B-cell receptor signaling and failures in tolerance [22].

## Clinical presentation

### Combined immunodeficiencies (CID)

The clinical manifestations of CID will vary depending on the specific underlying defect in the adaptive immune response. Therefore, clinical suspicion is important for timely diagnosis of these disorders. Patients may have lymphopenia (abnormally low levels of lymphocytes) and neutropenia (abnormally low levels of neutrophils). In the most severe forms of CID (also known as severe combined immunodeficiency [SCID]), there is a lack of T-cells and/or T-cell function. These disorders are rare and are generally categorized into whether there is an absence of T-cells, but presence of B-cells (T^─^, B^+^), or an absence of both T- and B-cells (T^−^, B^−^) (see Table 1). Natural killer (NK) cell numbers are also informative for determining the genetic phenotype of SCID [1, 2, 9]. However, normal T-cell numbers do not exclude the possibility of T-cell defects, and in patients with clinical presentations consistent with immunodeficiency, further investigations of T-cell function are warranted. Many CID have associated characteristics, such as thrombocytopenia or deoxyribonucleic acid (DNA) repair defects leading to malignancies, syndromic features (e.g., congenital anomalies, intellectual delay, hearing loss, short stature, ectodermal dysplasia) or dysmorphic facial features [23].

Patients with SCID may present within the first year of life with chronic diarrhea and failure to thrive, severe, recurrent infections with opportunistic pathogens (e.g., *Candida albicans* [thrush], *Pneumocystis jiroveci* [PJP], or cytomegalovirus [CMV]) and skin rashes. SCID is a pediatric emergency since infection often leads to death, and hematopoietic stem cell transplantation (HSCT) can be curative [2, 9].

Other, less severe CID that do not characteristically lead to early mortality include Wiskott-Aldrich syndrome, DiGeorge syndrome, and ataxia-telangiectasia. Patients with these disorders often present later in childhood with recurrent infections and clinical findings that vary depending on the specific syndrome (see Table 1). Autoimmunity and immune dysregulation are also frequent complications associated with these CID [2, 9].

Hyper-immunoglobulin E (IgE) syndrome is a CID characterized by staphylococcal infections of the skin, bone, and lungs, bony abnormalities and high IgE levels (see Table 1) [2, 9, 13, 14, 24]. It results from a mutation in signal transducer and activator of transcription 3 (*STAT3*) which affects phagocytic cell recognition of *Staphylococcus aureus* as well as osteoclast function involved in bone remodeling [24].

### Antibody deficiencies

Antibody deficiency disorders are the most commonly diagnosed immunodeficiency, accounting for approximately 50% of all IEI diagnoses [9, 25]. They comprise a heterogeneous group of disorders characterized by an increased susceptibility to respiratory tract infections with encapsulated bacteria, particularly *Streptococcus pneumoniae* and *Haemophilus influenzae.* Patients present after 6 months of age (after transplacentally transferred maternal IgG has gone) with recurrent, and often severe, sino-pulmonary infections such as otitis media, sinusitis, and pneumonia. Diarrhea, fatigue, autoimmune manifestations (particularly autoimmune cytopenias), hearing loss, and chronic lung disease (such as granulomas, bronchiectasis) are also common [26–29]. Patients with antibody deficiency often have reduced or absent serum Ig levels, but may also have normal or increased serum Ig levels with abnormal function. More than 50% of patients with antibody deficiency are diagnosed in adulthood [30] and there is generally a prolonged delay between first presentation and diagnosis since many healthcare providers do not consider IEI in their differential diagnosis.

More than 50 antibody-deficiency disorders have been genetically defined to date [1]. The best recognized/most common disorders in this category include: X-linked agammaglobulinemia (XLA; also known as Bruton’s agammaglobulinemia), common variable immunodeficiency (CVID), and selective IgA deficiency. XLA was the first described and genetically explored immunodeficiency disorder. It results from a mutation in the Bruton’s tyrosine kinase (*BTK*) gene, which is responsible for mediating B-cell development and maturation. The disorder is characterized by markedly reduced levels of circulating B-cells and serum IgG, IgA, and IgM. Affected males usually present within the first 2 years of life with recurrent sinopulmonary infections and absent lymph nodes and tonsils [9, 27]. CVID is a heterogeneous disorder characterized by markedly reduced serum concentrations of IgG, low levels of IgA and/or IgM, and poor or absent responses to immunization. The disorder affects males and females equally, and usually has a later age of onset than other antibody-deficiency disorders (e.g., > 10 years of age). It is associated with recurrent sinopulmonary infections, autoimmune and granulomatous disease, gastrointestinal complications, and an enhanced risk of malignancy (e.g., lymphoma and gastric carcinoma). Some patients may also present with bronchiectasis (irreversible widening of portions of the bronchi resulting from damage to the airway wall), which is a common cause of morbidity and mortality in these patients [9].

Milder antibody-deficiency disorders, such as selective IgA deficiency, are associated with variably low serum levels of an Ig class or subclass and, in some cases, impairments in specific antibody formation. Selective IgA deficiency, for example, is characterized by very low or absent levels of serum IgA in the presence of normal levels of IgG and IgM. Most patients with IgA deficiency are asymptomatic [31]. Among those who are symptomatic, up to one-third experience recurrent infections and one-third will present with organ-specific or systemic autoimmunity [9].

### Innate immunodeficiencies

Patients with innate immunodeficiency disorders may present at any age, often with unusual or difficult to eradicate infections. The typical signs and symptoms of phagocyte disorders are severe pyogenic (pus-like) bacterial and fungal infections of the skin, respiratory tract, and internal organs, as well as nail and gingival issues and painful sores around the mouth. Chronic granulomatous disease (CGD) is a phagocyte defect associated with a marked susceptibility to certain bacteria (catalase positive) and fungi.

Of all the IEIs, complement deficiencies account for 4% of identified cases. Patients with these disorders tend to present with systemic autoimmune disease that resembles lupus erythematosus or with severe or recurrent infections with encapsulated organisms (see Table 1) [2, 9, 13, 14, 24, 25].

### Disorders of immune dysregulation

These IEIs are associated with autoimmune disease due to the dysregulation of the immune system as a whole [32]. In many of these disorders, lymphocytes may be present but dysfunctional, allowing for the development of excessive autoreactivity and resultant autoimmune disease and/or other symptoms of immune dysregulation. Although the predominant manifestations are those of immune dysregulation (e.g., autoimmune cytopenia, lymphoproliferation, enteropathy, allergy, systemic autoimmunity, inflammation), infections can still occur. Disorders that fall into this category include: hemophagocytic lymphohistiocytosis (HLH), autoimmune lymphoproliferative syndrome (ALPS), immunodysregulation polyendocrinopathy enteropathy X-linked (IPEX), autoimmune polyendocrinopathy candidiasis and ectodermal dystrophy (APECED), and CTLA4 haploinsufficiency [19, 33–36].

### Primary atopic disorders

PAD are heterogeneous and have variable penetrance and expressivity. Patients with PAD can present with allergic inflammation solely or in combination with infections, autoimmunity, malignancy or immune dysregulation. Often, the allergic manifestations are severe and can be difficult to control, such as severe atopic dermatitis. Short stature, poor growth and connective tissue abnormalities have been observed in many PAD [21].

## Diagnosis

**Fig. 1:**
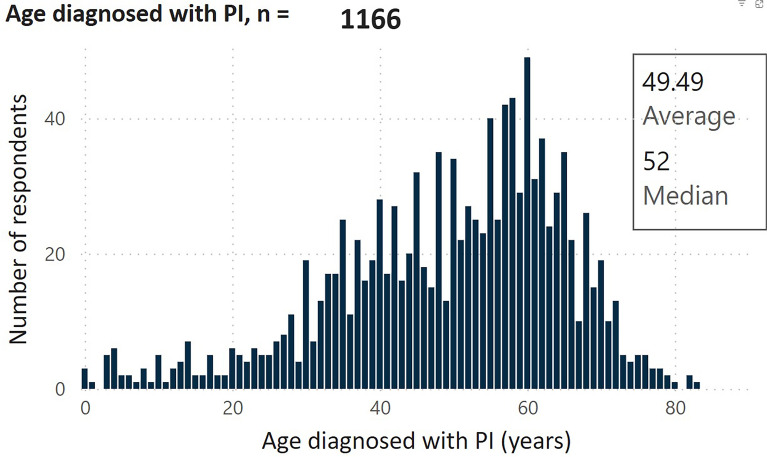
Results from the 2023 IDF national survey: Age of diagnosis with PI/IEI (n = 1166) [37]. IDF, Immune Deficiency Foundation; IEI, inborn errors of immunity; PID, primary immunodeficiencies. Figure courtesy of the Immune Deficiency Foundation (IDF). Figure is from the IDF 2023 National Patient Survey interactive report results available at: https://primaryimmune.org/advancing-pi-research-and-clinical-care/idf-surveys/2023-national-patient-survey Accessed November 19, 2024

**Fig. 2:**
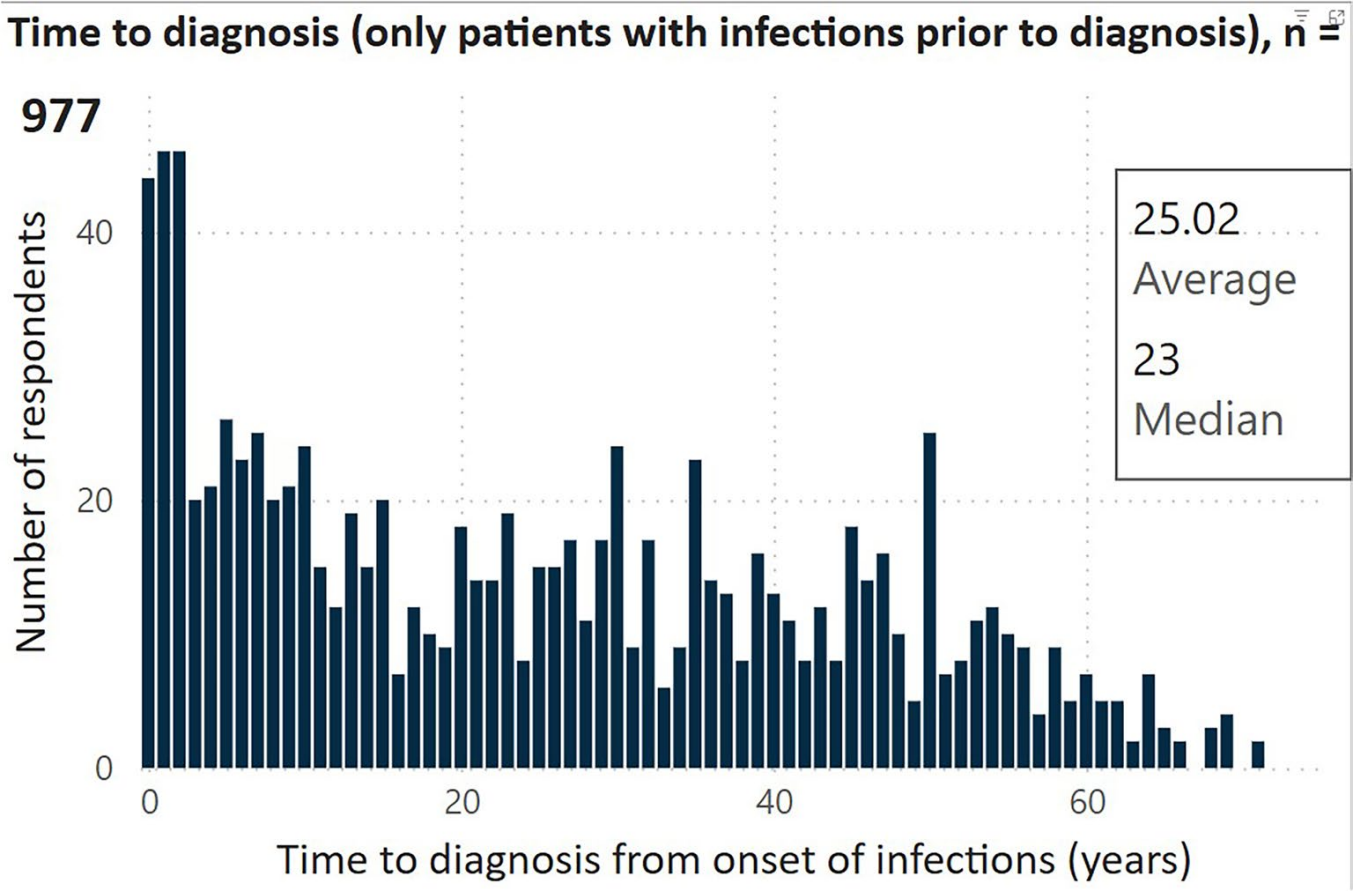
Results from the 2023 IDF national survey: Time to diagnosis (only patients with infections prior to diagnosis) (n = 977) [37]. PID, primary immunodeficiencies; IEI, inborn errors of immunity. Figure courtesy of the Immune Deficiency Foundation (IDF). Figure is from the IDF 2023 National Patient Survey interactive report results available at: https://primaryimmune.org/advancing-pi-research-and-clinical-care/idf-surveys/2023-national-patient-survey Accessed November 19, 2024

**Fig. 3:**
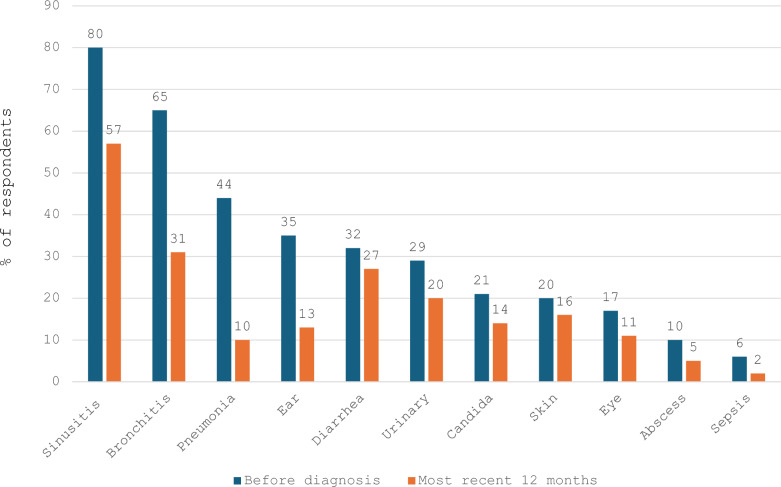
Results from the 2023 IDF national survey: Infections 12 months before diagnosis and most recent 12 months (n = 1177) [37]. Figure courtesy of the Immune Deficiency Foundation (IDF). Figure is adapted from the IDF 2023 National Patient Survey interactive report results available at: https://primaryimmune.org/advancing-pi-research-and-clinical-care/idf-surveys/2023-national-patient-survey Accessed November 19, 2024

Early diagnosis of IEI is critical for preventing significant disease-associated morbidity and mortality. However, national surveys conducted by the Immune Deficiency Foundation in the United States found that most patients with these disorders were not diagnosed until adulthood (mean 49.5 years) [37] (Fig. 1), with a mean time of 25 years from onset of infections to diagnosis (Fig. 2) [37]. The importance of prompt recognition and management of IEIs is further highlighted by the decrease in the percentage of individuals experiencing infections after diagnosis of immunodeficiency, compared to before diagnosis (Fig. 3) [37].

The difficulty in recognizing SCID in a timely fashion led to the application of newborn screening to this population. Briefly, when T-cell receptors are generated, a piece of DNA is cut out of the genome which is called a T-cell receptor excision circle (TREC). The TREC count can be used to quantify T-cell production in newborns and can identify many, but not all, cases of SCID. Infants identified through newborn screening receive expedited evaluation and, when needed, early HSCT [38]. Outcomes are dramatically improved and the risk of complications is reduced when HSCT is initiated early in SCID-affected infants, before the development of life-threatening infections or other complications [39, 40].

A diagnosis of IEI should be suspected in both children and adults who have recurrent pneumonias, ear, sinus, cutaneous and/or severe infections as listed in Table 2 [41] and severe atopic disorders [42] (see also Additional file 1: Supplementary appendix). Although Table 2 does not provide a comprehensive list of all signs and symptoms of IEI, patients meeting any of these criteria should be referred immediately to a clinical immunologist for further evaluation [10, 41]. Other important signs of IEI include excessive inflammatory responses such as severe atopy, autoimmunity, and cytopenias. It will also be important for the clinical immunologist to investigate for secondary causes of immunodeficiency, including medications, other infections, Ig loss and malignancy (see *Secondary Immunodeficiency* article in this supplement for more details).

**Table 2:**
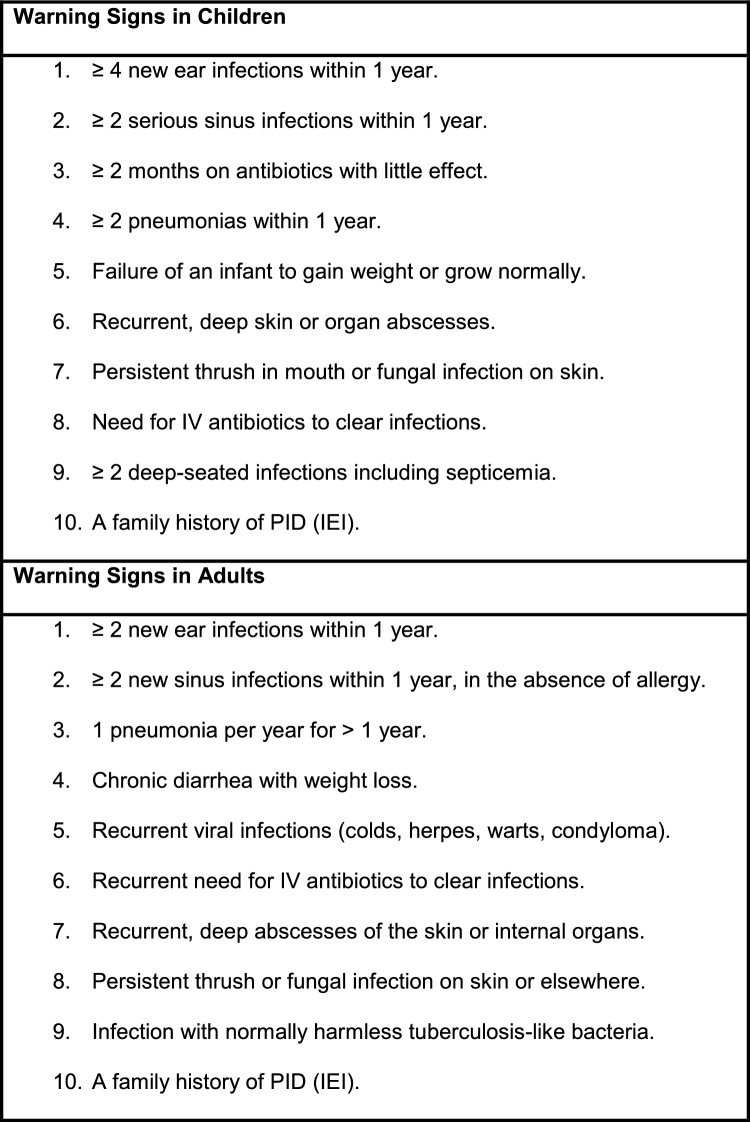
The Jeffrey Modell Foundation’s 10 warning signs of primary immune deficiency [41]

ADA, adenosine deaminase; AIRE: autoimmune regulator; ALPS, autoimmune lymphoproliferative syndrome; APECED, autoimmune polyendocrinopathy candidiasis and ectodermal dystrophy; CADINS, CARD11-associated atopy with dominant interference of NF-κB signaling; CVID, common variable immunodeficiency; HLH, hemophagocytic lymphohistiocytosis; IgA, immunoglobulin A; IgE, immunoglobulin E; IgG, immunoglobulin G; IFNγ, interferon-gamma; IL, interleukin; IPEX, immune dysregulation polyendocrinopathy enteropathy X-linked; JAK3, Janus kinase 3; LOF, loss of function; RAG, recombination activating gene; SCID, severe combined immunodeficiency; SLE, systemic lupus erythematosus; XLA, X-linked agammaglobulinemia

The immune evaluation often begins with a complete blood count (CBC) and blood smear. These tests are used to evaluate for the presence of lymphopenia, abnormal or unusual lymphocytes or phagocytic cells, and any associated gross hematologic abnormalities that may be indicative of IEIs. Significant lymphopenia, for example, may be the first indication of T-cell immunodeficiency. Flow cytometry can enumerate the numbers of B-cells, T-cells, and NK cells (e.g., lymphocyte subsets or immunophenotyping). Other important diagnostic tools include lymphocyte proliferation assays and the evaluation of lymphocyte markers, diversity of T cell receptors, and adhesion receptors that may be associated with specific immune defects. Standard flow cytometry analysis of lymphocyte subsets is abnormal in most cases of SCID and in many cases of CID [26, 43, 44].

The initial evaluation of patients with suspected antibody-deficiency disorders involves the measurement of serum IgG, IgA, and IgM levels (note that the measurement of IgD is not useful for the diagnosis of IEIs). Serum levels that are clearly below age-appropriate reference values may be indicative of B-cell immunodeficiencies. However, some patients with these disorders have normal or only modestly reduced Ig levels; therefore, the best approach for confirming a diagnosis of an antibody-deficiency disorder is the measurement of serum-specific antibody titers (usually IgG) in response to vaccine antigens. This approach involves immunizing a patient with protein antigens (e.g., tetanus toxoid) and polysaccharide antigens (e.g., pneumococcus) and assessing pre- and post-immunization antibody levels. In many IEIs, antibody responses to these antigens are diminished or even absent [26, 45]. However, interpretation of vaccination response may be challenging. A consensus document from the Basic and Clinical Immunology Interest Section of the American Academy of Allergy, Asthma and Immunology, developed in part using studies in healthy populations, can assist with the application of vaccine responses in the diagnosis of IEI [46].

Neutrophil function assays (e.g., dihydrorhodamine 1,2,3 response [DHR]) and stimulation assays for cytokine responses are helpful for confirming a diagnosis of innate disorders. For example, abnormal neutrophil oxidase function is usually indicative of CGD. Complement studies, which examine the level and/or function of specific complement proteins, are essential for the diagnosis of complement deficiency disorders. These studies should be performed by accredited laboratories that have demonstrated competence in these assays and experience in performing investigations into IEI [9, 26].

Advanced testing assessing the presence or function of cellular proteins often plays an integral role in the diagnostic evaluation. Furthermore, genetic testing, such as next-generation sequencing gene panel tests or whole exome sequencing, are already routinely used in complex or severe disease presentations of IEI [47–53]. A good example for its clinical value is demonstrated by the fact that in 25% of patients with the CVID phenotype, 70 identifiable gene defects may guide targeted treatment and personalized care [50, 51].

## Treatment

The treatment of IEIs is complex and generally involves both supportive and definitive strategies (see Table 3). As such, therapy often requires a multidisciplinary approach that should be coordinated by a clinical immunologist with expertise in the management of these disorders [9, 10]. It is important to consider that a genetic diagnosis is not required before initiation of treatment, as many patients with clinical and laboratory evidence of IEI may not, as of yet, have an identified single gene defect [43, 44]. Thus, therapy should be initiated based on the patient’s clinical and laboratory status. Delays in treatment can lead to permanent organ damage or even death from overwhelming infection [9]. A genetic diagnosis, however, might explain the immune pathophysiologic mechanism and guide targeted treatment, such as immunosuppressive therapies, including small-molecule inhibitors, biologic therapies, immune modulators [54], gene therapy, and/or HSCT. For children and adults with a molecular diagnosis of IEI, additional considerations exist for immunization strategies. Some vaccines may be contraindicated, others may require additional doses compared to healthy individuals, or may require a specific timing of administration in relation to treatment options being considered [55].

**Table 3:**
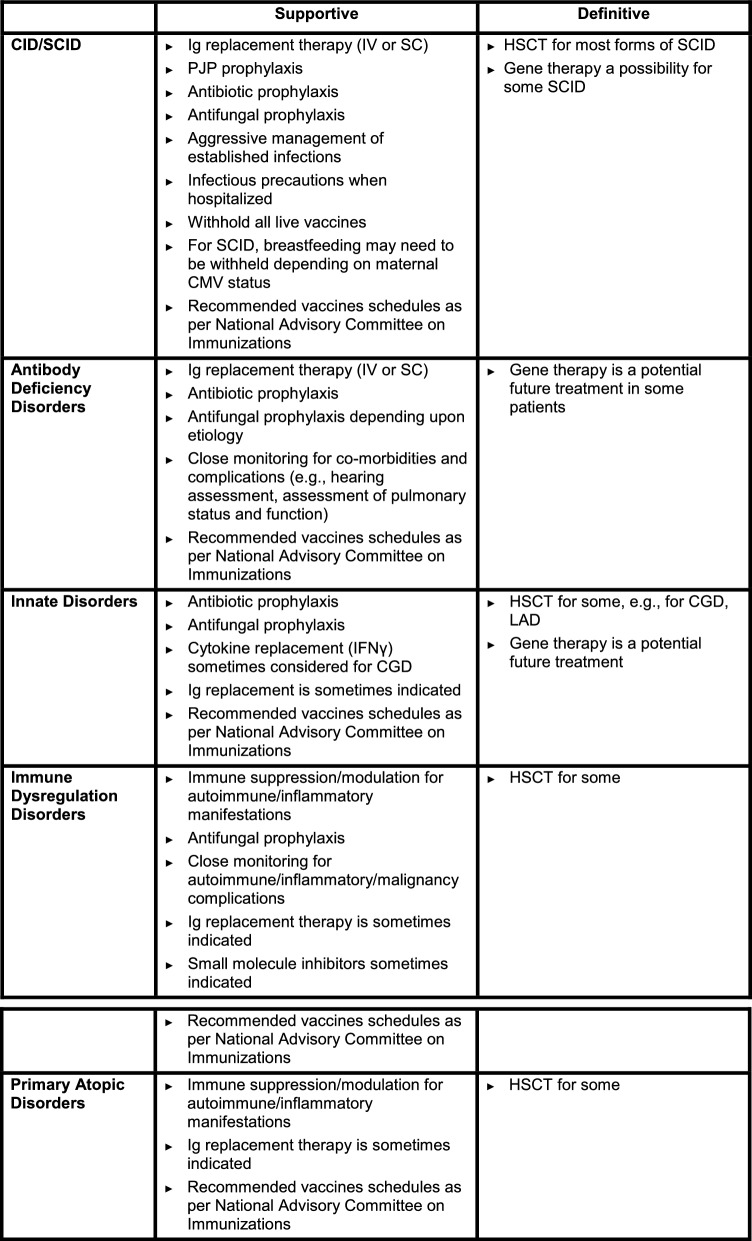
Strategies for the treatment and management of IEIs

CGD, chronic granulomatous disease; CID, combined immunodeficiency; HSCT, hematopoietic stem cell transplantation; IFNγ, interferon-gamma; Ig, immunoglobulin; IV: intravenous; LAD: leukocyte adhesion defect; PJP, *Pneumocystis jiroveci* pneumonia; SC, subcutaneous; SCID, severe combined immunodeficiency

### SCID/CID

Initial therapy for patients with SCID or other CID is supportive and involves aggressive treatment of the established infection, Ig replacement therapy (discussed in more detail in the next section), and antibiotic, antiviral, and/or antifungal prophylaxis to reduce the frequency and severity of infections. There is currently no standardized approach to the use of prophylactic antimicrobials in patients with established IEIs since randomized, controlled studies in this area are lacking. Commonly used regimens are derived from studies focusing on the prevention of otitis media in children and include: amoxicillin, trimethoprim-sulfamethoxazole (TMP-SMX) and azithromycin [2, 9]. Patients with SCID should start prophylaxis against PJP. They should also be protected from exposure to infectious agents, such as limiting exposures to others, avoidance of breast-feeding in CMV-positive mothers and ensuring that blood products are irradiated and CMV-negative. Furthermore, live attenuated vaccines (e.g., measles/mumps/rubella/varicella, bacillus Calmette-Guerin, infant rotavirus, oral polio virus) are contraindicated in patients with SCID as they can lead to severe, disseminated, and fatal infections [9, 55]. There is no risk of disseminated infections from killed or inactivated vaccines and, therefore, these may be administered according to routine indications and schedules in patients with CID, recognizing that the immune protection gained from these vaccines may be suboptimal. Immediate family members should also ensure that their immunization status is up to date; however, some live vaccines such as oral polio, smallpox and live attenuated influenza vaccines should be avoided due to risk of transmission to the immunocompromised individual [55].

Since SCID is fatal unless the underlying defect is corrected, definitive therapy with HSCT should be initiated as quickly as possible. When performed from a human leukocyte antigen (HLA)-identical sibling, these procedures lead to excellent long-term survival and long-lasting immune reconstitution. Improvements in HLA typing, conditioning regimens and supportive therapies have led to improved outcomes after HSCT for alternative donor sources, such as HLA-matched unrelated donors, haploidentical related donors and umbilical cord donors [2, 9, 56–58].

Gene therapy, which involves introducing a functional copy of the patient's defective gene into appropriate cells, has also been shown to lead to immune reconstitution and improved survival in patients with certain forms of IEI, such as adenosine deaminase (ADA) deficiency-SCID, SCID-X1 (an X-linked inherited SCID characterized by an early block in T-cell differentiation), Wiskott-Aldrich syndrome and CGD [59–63]. There are challenges with safety, access and effectiveness and its use is currently primarily in the research domain [64]. Enzyme replacement therapy with weekly intramuscular injections of recombinant ADA is also available for the management of patients with ADA deficiency [65].

### Antibody deficiency disorders

The mainstay of therapy for most antibody-deficiency disorders is intravenous (IV) or subcutaneous (SC) Ig replacement therapy and immunoprophylaxis with vaccines; in fact, many patients will require this treatment lifelong. There are now several sources available for gammaglobulin licensed by Health Canada for use in patients with IEI. Table 4 lists some of the IV and SC Ig products carried by Canadian Blood Services [66], and Table 5 lists the Ig products carried by Héma-Québec [67]. However, it is important to note that these products may not be available in all cities/provinces in Canada, and that other products not listed in this table may be available for the treatment of IEI. IV and SC formulations are considered equally effective in reducing the frequency and severity of infections, and there is insufficient evidence to suggest that one product is superior to another, although dosing and frequency of use must be carefully monitored [9, 10, 68]. When deciding on a specific product, patient preference should be taken into consideration [69]. Many patients prefer a SC formulation since therapy can be administered at home. A recently approved hyaluronidase facilitated SC Ig product (fSC Ig) allows less frequent and greater volume infusions [70]. Note that intramuscular Ig replacement therapy is not considered to be as effective as IV or SC therapy and, therefore, is not recommended for the treatment of IEI.

**Table 4:**
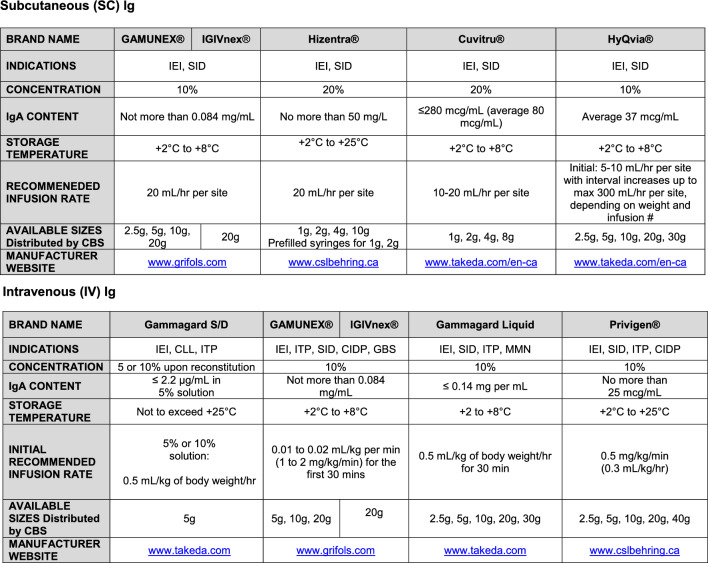
Subcutaneous and intravenous Ig products carried by Canadian Blood Services [66]

IEI, inborn errors of immunity: SID: secondary immunodeficiency; ITP: idiopathic thrombocytopenic purpura; CIDP: chronic inflammatory demyelinating polyneuropathy; CLL: B-cell chronic lymphocytic leukemia; MMN: multifocal motor neuropathy

Adapted from Canadian Blood Services 2018. Complete tables available at: https://professionaleducation.blood.ca/en/transfusion/clinical-guide/immune-globulin-products

These products may not be available in all cities/provinces across Canada, and other Ig products not listed here may be available

**Table 5:**
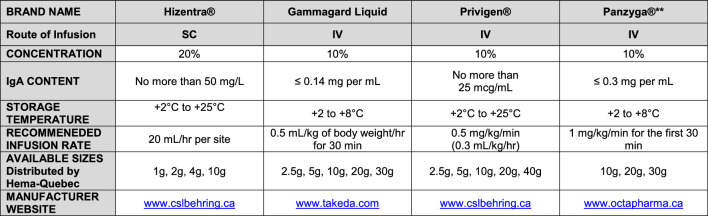
Subcutaneous and intravenous Ig products carried by Hema-Quebec

^**^product will be discontinued once inventory runs out

Adapted from Héma-Québec (2024). Complete tables available at: https://www.hema-quebec.qc.ca/sang/professionnels-sante/produits-sanguins-stables/index.en.html

These products may not be available in all cities/provinces across Canada, and other Ig products not listed here may be available

Most patients with antibody-deficiency disorders, such as CVID or XLA, are not able to mount significant responses to immunizations. Administration of non-live vaccines may thus be futile, although not harmful. Patients receiving regular Ig replacement therapy should not receive live viral vaccines as Ig can interfere with the immune response to some live attenuated viral vaccines [55].

The recommended starting dose of Ig replacement therapy is 400–600 mg/kg/dose given every 3–4 weeks for the IV formulation, 100–150 mg/kg/dose given 1–2 times per week for SC Ig, and 300–800 mg/kg given every 3–4 weeks for fSC Ig [10, 71]. The most common adverse events associated with this therapy are headache, flushing, chills, myalgia, wheezing, tachycardia, lower back pain, nausea, and hypotension, that may be decreased or prevented with premedication using antipyretics, antihistamines or corticosteroids. In patients experiencing multiple adverse reactions to one product, consideration may be given to switching to another product or route of administration [10, 71]. Evidence suggests that trough (IV Ig) or steady-state (SC Ig) IgG levels should be assessed regularly, and that dose may need to be adjusted depending upon the frequency of infection. Lower trough levels have been associated with the progression of chronic lung disease in otherwise asymptomatic patients [68, 72], suggesting that physicians must be diligent in maintaining good levels of serum IgG, and should increase the amount given if there are signs of changing lung function or if the patient continues to experience recurrent infections.

For patients with recurrent infections, prophylactic antibiotic therapy (particularly with agents that provide coverage of *Streptococcus pneumoniae* and *Haemophilus influenzae)* may also be needed in addition to Ig replacement therapy. Azithromycin prophylaxis has been shown to reduce the frequency of respiratory exacerbations and reduce the use of antibiotics in patients with antibody deficiency [73]. Depending on the etiology of the specific B-cell disorder, prophylactic antifungal therapy may also be required. Since B-cell immunodeficiencies are often associated with hearing loss and pulmonary complications, regular hearing assessments and monitoring of pulmonary status and function is recommended. As with primary T-cell defects, vigilance for malignancies and autoimmune disorders is also important in patients with B-cell disorders.

At present, there are no definitive management strategies that can be routinely recommended for patients with B-cell disorders. However, gene therapy is currently being investigated for some antibody deficiencies and may represent a future treatment option for these patients [59, 60].

### Innate disorders

The management of innate disorders depends on the type of defect. For phagocyte disorders, therapy is primarily supportive and includes both antibiotic and antifungal prophylaxis. The use of cytokine replacement (e.g., interferon-gamma) as routine prophylaxis is controversial as studies demonstrating its efficacy were largely done before the use of routine anti-fungal prophylaxis [74, 75]. HSCT is an effective curative treatment option for patients with CGD [76]. Gene therapy may also be a potential definitive treatment option in the future [9, 59, 60, 63].

There is no specific definitive therapy for complement deficiencies. Management of these disorders focuses on antibiotic prophylaxis for the prevention of recurrent infections and vaccination. Since some patients with complement disorders are at increased risk of meningococcal infections with *Neisseria meningitidis,* multivalent meningococcal vaccinations should also be considered (see the *Canadian Immunization Guide for the vaccination of immunocompromised patients with primary immune deficiencies***)** [55]. Pneumococcal and *Haemophilus influenzae* vaccines should also be considered in patients with frequent infections caused by encapsulated organisms.

### Immune dysregulation

Many patients with IEI have autoimmune or inflammatory complications of immune dysregulation, requiring immune suppression and/or modulation [28, 77]. Patients should be actively monitored for such manifestations. Treatment usually requires a multidisciplinary approach, as immune suppression can increase the risk of infection in already immunocompromised patients. The IEI diagnosis and/or underlying pathophysiology may be helpful in determining the most appropriate treatment. For example, patients with IPEX (immune dysregulation, polyendocrinopathy, enteropathy, X-linked) syndrome have impaired Treg function, leading to significant autoimmunity. Treatment with rapamycin, an inhibitor of mammalian target of rapamycin (mTOR), has been shown to improve Treg function and autoimmune manifestations in patients with IPEX syndrome and stabilize their state prior HSCT [78, 79].

## Prognosis

The prognosis of patients with IEIs varies depending on the etiology of the disorder. However, patient outcomes and long-term survival have improved significantly since the 1970s given improvements in recognition and diagnosis, management of infections, early access to antimicrobials, advances in HSCT techniques, and enhanced intensive care services [25, 80]. Furthermore, routine vaccinations provide herd immunity to those at risk, decreasing the circulation of infectious diseases. Further progress in the diagnosis and management of IEIs is expected as research on the genes responsible for immunodeficiencies and the use of definitive treatments such as gene therapy continues.

## Conclusions

IEI are a heterogeneous group of disorders that result from defects in immune system development and/or function. IEIs present in both children and adults, and although signs and symptoms are highly variable, most disorders involve increased susceptibility to infection, with many leading to significant disease-associated morbidity and mortality. Other important signs of IEI include excessive inflammatory responses, primary atopic disorders, autoimmunity and cytopenias. Given the complexity of these disorders, referral to a clinical immunologist is required for appropriate diagnosis and management. Severe disorders such as SCID require definitive therapy for immune reconstitution (e.g., HSCT, gene therapy) as soon as possible, which has led to the application of newborn screening to this population. Antibody-deficiency disorders are the most common types of IEIs. The mainstay of treatment for patients with these disorders is Ig replacement therapy. For all IEIs, attention must be given to immunization schedules which can vary from healthy individuals regardless of age. Physicians must be diligent in maintaining good levels of serum IgG since lower trough levels have been associated with the progression of chronic lung disease in otherwise asymptomatic patients. Patients with innate immunodeficiency disorders often present with unusual or difficult to eradicate infections. Treatment varies depending on the type of defect (e.g., phagocyte disorder or complement deficiency), but may involve antifungal and antibiotic prophylaxis, cytokine replacement, immunomodulation, vaccinations and HSCT.

## Supplementary Information

Additional file 1.

## Data Availability

Not applicable.

## Abbreviations

ADA: Adenosine deaminase
AIRE: Autoimmune regulator
ALPS: Autoimmune lymphoproliferative syndrome
APECED: Autoimmune polyendocrinopathy candidiasis and ectodermal dystrophy
BTK: Bruton’s tyrosine kinase
CBC: Complete blood count
CGD: Chronic granulomatous disease
CID: Combined immunodeficiencies
CMV: Cytomegalovirus
CVID: Common variable immunodeficiency
DHR: Dihydrorhodamine 1,2,3 response
DNA: Deoxyribonucleic acid
HCST: Hematopoietic stem cell transplantation
HLA: Human leukocyte antigen
HLH: Hemophagocytic lymphohistiocytosis
IEI: Inborn error of immunity
IFNγ: Interferon-gamma
IL: Interleukin
Ig: Immunoglobulin
IPEX: Immune dysregulation polyendocrinopathy enteropathy X-linked
IV: Intravenous
JAK3: Janus kinase 3
mTOR: Mammalian target of rapamycin (mTOR)
NK: Natural killer
PAD: Primary atopic disorders
PJP: *Pneumocystis* *jiroveci* Pneumonia
RAG: Recombination activating gene
SC: Subcutaneous
SCID: Severe combined immunodeficiency
SLE: Systemic lupus erythematosus
STAT3: Signal transducer and activator of transcription 3
TLR: Toll-like receptors
TMP-SMX: Trimethoprim-sulfamethoxazole
TREC: T-cell receptor excision circle
XLA: X-linked agammaglobulinemia
