# Supplementary material for: Inborn errors of immunity (primary immunodeficiencies)
Source: Allergy Asthma Clin Immunol. 2025 Jan 8;20(Suppl 3):76. doi: 10.1186/s13223-024-00938-z (PMC11714877; doi:10.1186/s13223-024-00938-z)
Supplement: Supplementary file 1 — Additional file 1. [file 13223_2024_938_MOESM1_ESM.pdf]

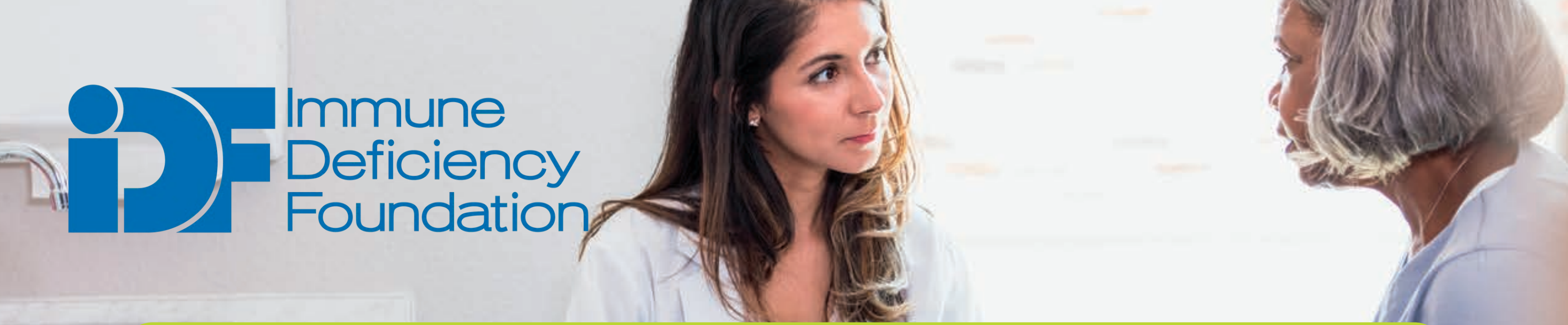

Immune Deficiency Foundation

Are you missing patients with PRIMARY IMMUNODEFICIENCY?

Primary immunodeficiencies (PIs), also known as inborn errors of immunity (IEI), are a group of 450+ rare and chronic disorders caused by genetic variants. Patients average 9-15 years from symptom onset to diagnosis and experts estimate that more than 70% of individuals with a PI remain undiagnosed.

DOES YOUR PATIENT HAVE...

- Infections that are frequent, persistent, unusual, and/or difficult to treat?
- Unusual complications of common infections?
- Unusual autoimmunity?
- Enlarged lymph nodes that persist and/or hepatosplenomegaly?
- Non-healing skin ulcers?
- A failure to thrive or unintentional weight loss?
- Recurrent fevers with no source?

OTHER SIGNS & SYMPTOMS

PIs are not just pediatric disorders; ~75% of patients are diagnosed as adults.

Not all PIs are life-threatening, many are mild/moderate and patients don't 'look' sick.

Immune
Deficiency
Foundation

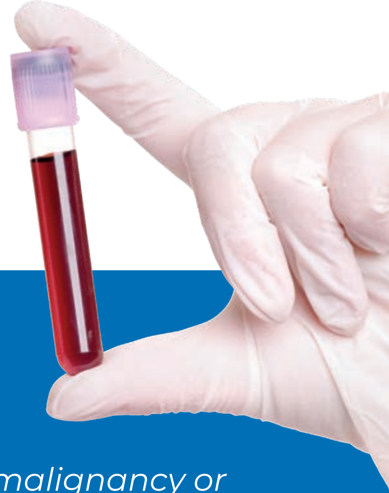

START A DIAGNOSTIC WORKUP

1. Collect detailed patient history, including family history.

Patient history: infections (site, organism, frequency, and severity), any malignancy or lymphoproliferation, frequency and severity of any autoimmune or autoinflammatory symptoms, failure to thrive or unintentional weight loss. Family history: diagnosed PI, early childhood deaths, family members with PI-like symptoms.

2. Order complete blood count with differential and compare absolute counts of lymphocytes, neutrophils, eosinophils, and monocytes to age-adjusted reference ranges.

3. Order serum immunoglobulin (Ig) test to assess the patient's overall IgG, IgA, and IgM levels, and antibody titers to vaccine antigens.

Some patients with PI lack functional antibodies, which can be masked by normal overall serum Ig levels. Titers to tetanus toxoid and/or pneumococcal vaccine measure specific antibody response.

4. Consider referral to a clinical immunologist even if initial labs appear normal.

HEALTHCARE PROVIDER RESOURCES

SCAN ME

CLINICIAN FINDER

is a free tool that lists clinicians of any specialty with experience treating patients with PI. Listings can be filtered by location and specialty. To be added to the Clinician Finder, contact IDF.

CONSULTING IMMUNOLOGIST PROGRAM

is a free clinician-to-clinician service that provides a second opinion or consultation from a national network of renowned clinical immunologists specializing in PI.

The Immune Deficiency Foundation (IDF) improves the diagnosis, treatment, and quality of life of people affected by primary immunodeficiency through fostering a community empowered by advocacy, education, and research.
